# First detection of a plasmid-encoded New-Delhi metallo-beta-lactamase-1 (NDM-1) producing *Acinetobacter baumannii* using whole genome sequencing, isolated in a clinical setting in Benin

**DOI:** 10.1186/s12941-020-00411-w

**Published:** 2021-01-06

**Authors:** Carine Yehouenou, Bert Bogaerts, Kevin Vanneste, Nancy H. C. Roosens, Sigrid C. J. De Keersmaecker, Kathleen Marchal, Dissou Affolabi, Reza Soleimani, Hector Rodriguez-Villalobos, Françoise Van Bambeke, Olivia Dalleur, Anne Simon

**Affiliations:** 1 Clinical Pharmacy Research Group (CLIP), Louvain Drug Research Institute (LDRI), Université Catholique de Louvain UCLouvain, Brussels, Belgium; 2 Laboratoire de Référence des Mycobactéries (LRM), Cotonou, Benin; 3 Faculté des Sciences de la Santé (FSS), Université d’Abomey Calavi (UAC), Cotonou, Benin; 4 Sciensano, Transversal Activities in Applied Genomics, Brussels, Belgium; 5 Centre National Hospitalier et Universitaire Hubert Koutoukou Maga (CNHU-HKM) Country Cotonou, ., Benin; 6 Pharmacy, Clinique Universitaire Saint-Luc, Université Catholique de Louvain, UCLouvain, Brussels, Belgium; 7 Microbiologie, Cliniques Universitaires Saint Luc, Université Catholique de Louvain, UCLouvain, Brussels, Belgium; 8 Pole de Microbiologie, Institut de Recherche Expérimentale et Clinique (IREC), Université Catholique de Louvain UCLouvain, Brussels, Belgium; 9 Department of Plant Biotechnology and Bioinformatics, Ghent University, Ghent, Belgium; 10 Department of Information Technology, IDLab, Ghent University, IMEC, Ghent, Belgium; 11 Pharmacologie Cellulaire et Moléculaire, Louvain Drug Research Institute (LDRI), Université Catholique de Louvain UCLouvain, Brussels, Belgium

**Keywords:** *Acinetobacter baumannii*, New-Delhi metallo-beta-lactamase, Whole-genome sequencing, Benin

## Abstract

**Background:**

Carbapenem-resistant *Acinetobacter baumannii* is considered a top priority pathogen by the World Health Organization for combatting increasing antibiotic resistance and development of new drugs. Since it was originally reported in *Klebsiella pneumoniae* in 2009, the quick spread of the *bla*_NDM-1_ gene encoding a New-Delhi metallo-beta-lactamase-1 (NDM-1) is increasingly recognized as a serious threat. This gene is usually carried by large plasmids and has already been documented in diverse bacterial species, including *A. baumannii*. Here, we report the first detection of a NDM-1-producing *A. baumannii* strain isolated in Benin.

**Case presentation:**

A 31-year-old woman was admitted to a surgical unit with a diagnosis of post-cesarean hematoma. An extensively-drug resistant *A. baumannii* strain solely susceptible to amikacin, colistin and ciprofloxacin, and resistant to several other antibiotics including ceftazidime, imipenem, meropenem, gentamicin, tobramycin, ceftazidime/avibactam, and sulfamethoxazole-trimethoprim, was isolated from the wound. Production of NDM-1 was demonstrated by immunochromatographic testing. Whole genome sequencing of the isolate confirmed the presence of *bla*_NDM-1_, but also antibiotic resistance genes against multiple beta-lactamases and other classes of antibiotics, in addition to several virulence genes. Moreover, the *bla*_NDM-1_ gene was found to be present in a Tn125 transposon integrated on a plasmid.

**Conclusions:**

The discovery of this extensively-drug resistant *A. baumannii* strain carrying *bla*_NDM-1_ in Benin is worrying, especially because of its high potential risk of horizontal gene transfer due to being integrated into a transposon located on a plasmid. Strict control and prevention measures should be taken, once NDM-1 positive *A. baumannii* has been identified to prevent transfer of this resistance gene to other Enterobacterales. Capacity building is required by governmental agencies to provide suitable antibiotic treatment options and strategies, in combination with strengthening laboratory services for detection and surveillance of this pathogen.

## Background

*Acinetobacter baumannii* is an opportunistic nosocomial pathogen responsible for a broad range of infections [1]. Nosocomial isolates of this bacterium are often resistant to almost all currently available antibiotics. Some striking features of this bacterium, such as its ability to cause opportunistic infections, to develop antimicrobial resistance and to survive under adverse environmental conditions, have contributed to its wide dissemination [1]. The global spread of carbapenem-resistant *A. baumannii* has been observed and is considered a sentinel event of emerging antimicrobial resistance [2].

Carbapenem resistance mechanisms in *A. baumannii* are more commonly mediated by carbapenem-hydrolyzing class D beta-lactamases and less often by class B metallo-beta-lactamases [3]. Gram-negative bacteria with the New-Delhi metallo-beta-lactamase type-1 (NDM-1), encoded by the *bla*_NDM-1_ gene, utilize at least one zinc atom at the active site to facilitate hydrolysis of a broad variety of beta-lactams and carbapenems [4]. The initial case detection of NDM-1 production was reported in 2009 in a clinical urinary isolate of *Klebsiella pneumoniae* from a 59-year-old man who returned to Sweden after hospitalization in India [5]. Afterwards, the genetic context of *bla*_NDM-1_ was reported in 2011 in a clinical *A. baumannii* isolate discovered in a German hospital [6]. The *bla*_NDM-1_ gene was integrated on a new transposon structure (Tn125) flanked by two insertion elements [6]. Subsequently, Tn125-harboring *bla*_NDM-1_ was reported on chromosomes in several multiple-resistant *Acinetobacter* spp. isolates throughout Europe [7]. Moreover, a *bla*_NDM-1_ bearing plasmid, pNDM-BJ01, was reported in 2012, isolated from a clinical *Acinetobacter lwoffii* strain in China [8]. This plasmid carried a complete Tn125 transposon and showed high horizontal transferability. Since then, several NDM-1 positive plasmids have been isolated from *Acinetobacter* spp. in China with similar structures to pNDM-BJ01 [9]. To date, NDM carbapenems have been reported in most regions around the world owing to the rapid dissemination of the gene between members of the Enterobacterales and *Acinetobacter* spp. in human and environmental isolates [1]. So far, only limited reports exist on NDM-1 producing *A. baumannii* in Africa, and those published are mostly from northern or southern African countries [10, 11]. Here, we describe the first case of an extensively drug-resistant *A. baumannii* strain producing NDM-1- in Benin.

## Case presentation

A 31-year-old woman was admitted to a surgical unit at a public hospital in Benin on March 06, 2019. She had undergone a ceasarean in a different hospital and presented hematoma, but previous treatment information and conditions were not recorded upon admission. During laparotomy, surgical antimicrobial prophylaxis was administered as intravenous ceftriaxone (1 g), and after intervention, empirical antibiotic therapy consisting of intravenous imipenem (500 mg every 8 h) was initiated for one week. On the tenth day, the patient’s clinical condition worsened and she developed fever (38 °C) and wound suppuration. Preliminary investigation revealed that the patient had no previous history of travel or hospitalization abroad. Intensive programs of environmental cleaning and strict contact isolation precautions were applied. However, following the initial treatment of 500 mg imipenem per 8 h, the patient preferred to continue with unspecified indigenous treatment due to lack of financial support. As she was no longer in the hospital, the clinical outcome is unknown.

The culture of a pus swab revealed Gram-negative coccobacilli that were glucose-non-fermentative, non-motile, and oxidase-negative. Biochemical identification was performed with the Analytical Profile Index (API 20E, Biomérieux, France) and results were confirmed by matrix-assisted laser desorption/ionization time-of-flight (MALDI-TOF) mass spectrometry. Antimicrobial susceptibility testing was assessed using the modified Kirby-Bauer disc diffusion method and confirmation was done by the microbroth dilution method. The interpretation breakpoints were based on the criteria of the European Committee on Antimicrobial Susceptibility Testing (EUCAST) (http://www.eucast.org/ast_of_bacteria/). Except for amikacin, colistin and ciprofloxacin, the isolate was resistant to all tested antimicrobial agents with the following Minimum Inhibitory Concentration (MIC) values: ceftazidime (> 16 mg/l), imipenem and meropenem (> 16 mg/l), gentamicin and tobramycin (> 8 mg/l), ceftazidime/avibactam (> 16/4), sulfamethoxazole-trimethoprim (> 8/152). The isolate was therefore considered as extensively drug resistant (XDR) [4]. Additionally, to confirm the resistance pattern, we used the multiplex lateral flow immunochromatographic test, the RESIST-3 O.K.N. ICT (Coris Bioconcept, Gembloux, Belgium), which confirmed the presence of NDM.

Whole-genome sequencing (WGS) was subsequently performed for detection and characterization of resistance genes, using DNA from a single-colony isolate employing the EZ1 advanced XL biorobot and the tissue DNA kit (Qiagen, Hilden, Germany) with the bacterial card, according to the manufacturer’s instructions. A standard Nextera XT library (Illumina, San Diego, USA) was constructed (Nextera XT DNA library preparation kit, Illumina, San Diego, USA) and subsequently sequenced on an Illumina MiSeq instrument with a 250-bp paired-end protocol (MiSeq v3 chemistry, Illumina, San Diego, USA) according to the manufacturer’s instructions. Data was analyzed as follows. First, reads were trimmed with Trimmomatic 0.36 [12] with the settings ‘NexteraPE-PE.fa:2:30:10’, ‘LEADING:10’, ‘TRAILING:10’, ‘SLIDINGWINDOW:4:20’, and ‘MINLEN:40’. Processed reads were then assembled de novo using SPAdes 3.13.0 [13] with the ‘careful’ option enabled and the ‘cov-cutoff’ parameter set to 10. Contigs smaller than 1000 bases were removed with seqtk seq 1.2 (https://github.com/lh3/seqtk) using the ‘-L’ option. Assembly statistics were determined using Quast 4.4 with default settings [14]. Genome annotations were created using Prokka 1.13 [15] (Table 1).

**Table 1:** Genome assembly, and putative plasmid, statistics

	Cumulative length (bp)	Nb. of contigs	GG-content (%)	Nb. of coding sequences	Nb. of tRNAs	NCBI accession
Genome assembly	4,082,860	39	38.92	3838	64	JABBFT000000000
Putative plasmid	88,063	1	42.13	101	0	JABBFT000000000 (Seqeuence ID: Sciensano:GLGAKIBC_35)

This table contains statistics for the de novo assembly and annotation of the genome and putative plasmid. The number of coding sequences and tRNAs were extracted from the Prokka annotations. The genome and putative plasmid sequences were uploaded to NCBI as a single submission, with the putative plasmid annotated as ‘Sciensano:GLGAKIBC_35’

The NCBI National Database of Antibiotic Resistant Organisms (NDARO) [16], Virulence Factor (full) database (VFDB) [17], and PlasmidFinder database, were used for genotypic detection of genes encoding antimicrobials, virulence factors, and plasmid replicons, respectively, using SRST2 0.2.0 [18] with default settings. The XDR status of the isolate was confirmed by harboring several resistance genes against aminoglycosides (*aph(6)*-*Id*, *aph(3”)*-*Ib, ant(3’’)*-*IIa,* and *aac(3)*-*IId*), beta-lactamases (*bla*_NDM-1_, *bla*_OXA-58_, *bla*_OXA-558_, *bla*_ADC-166_), macrolide-lincosamide-streptogramin B (*msr(E)*), macrolide (*mph(E)*), sulfonamide (*sul2*), tetracycline (*tet(39)*), and bleomycin (*ble*). Additionally, 67 loci encoding different virulence factors were detected including genes related to biofilm formation such as *ompA*, *bfmS*, *csuE*, and a K1 capsular polysaccharide (ABK1) (see Additional file 1). No plasmid replicons from the PlasmidFinder database were detected.

**Fig. 1:**
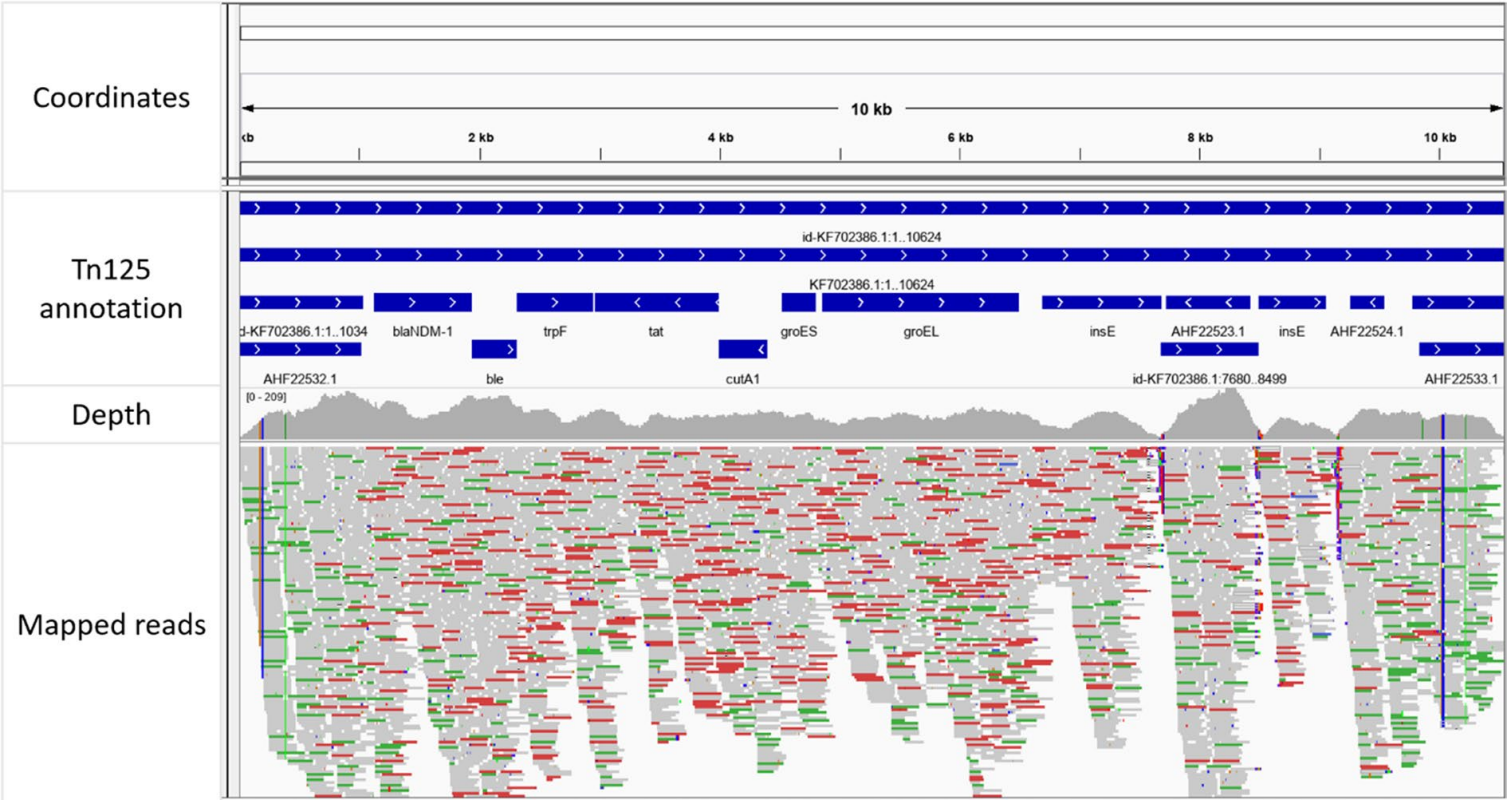
Detection of the Tn125 transposon carrying the *bla*_NDM-1_ gene. The top half of the figure contains the genomic coordinates and annotation of the Tn125 transposon sequence retrieved from NCBI (KF702386.1). The lower half of the figure contains the sequencing depth and reads mapped to the corresponding regions. Visualization created with IGV [29]

**Fig. 2:**
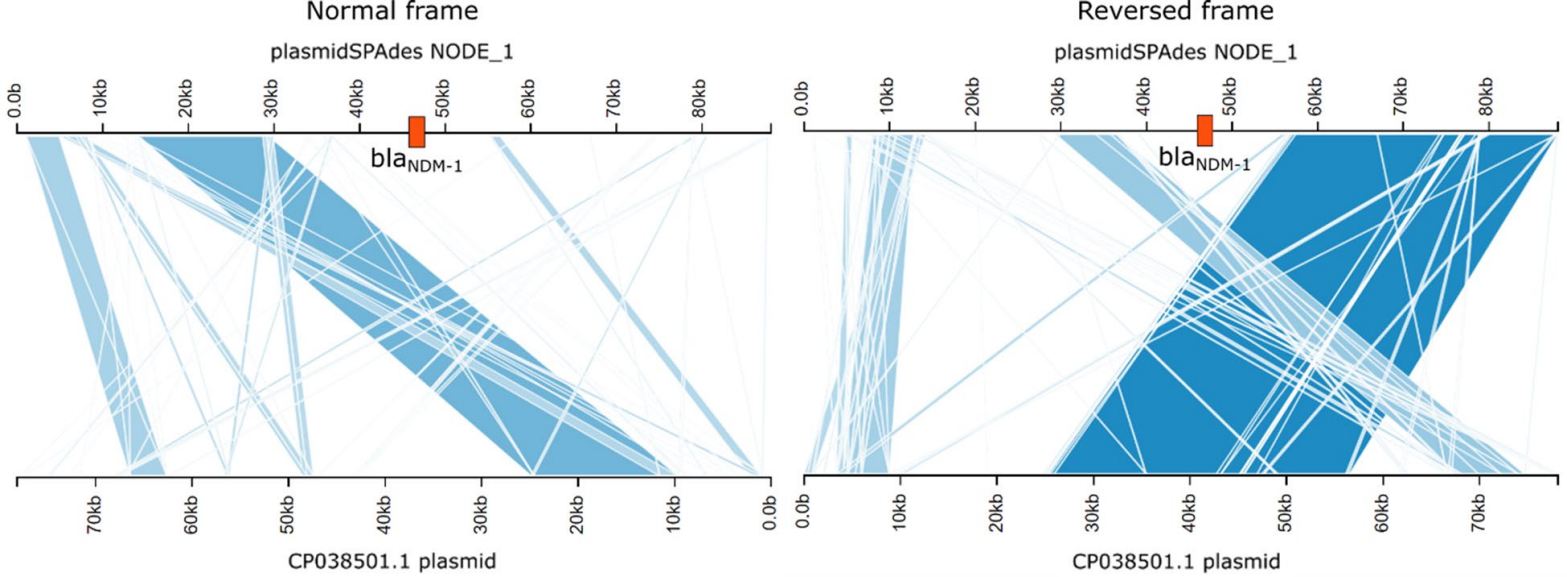
Alignment of the putative plasmid containing *bla*_NDM-1_ against the CP038501.1 plasmid. The figure illustrates the putative plasmid generated with plasmidSPAdes aligned to the CP038501.1 plasmid that was detected as best match with PLSDB. The location of *bla*_NDM-1_ on the putative plasmid is indicated in orange. High-quality alignments between the putative plasmid and CP038501.1 are indicated in blue (with darker blue representing higher BLAST bit scores). The left and right plots illustrate the alignments in normal frame (plus strand/plus strand) and reverse frame (plus strand/minus strand), respectively. Visualization created with Kablammo [30]

Sequence typing was performed with the corresponding regular multi-locus sequence typing (MLST) schemes from Oxford University and Institut Pasteur [19], as described in Bogaerts et al. [20]. MLST analysis detected sequence type 836 (Oxford University scheme) and 388 (Institut Pasteur scheme). No isolates were present for the former, but the latter returned two isolates from Taiwan (from 2012 and 2013) and a single isolate from Norway (year unknown) with the same sequence type. The sample was screened for the presence of the Tn125 transposon by mapping (trimmed) reads against its reference sequence (NCBI KF702386.1) using Bowtie2 2.3.0 [21] with the ‘–sensitive’ setting enabled. In total, 99.55% of the Tn125 transposon reference length of 10,624 bp was covered by at least one read, with three breakpoints however present at ~ 7.7 kb, 8.5 kb, and 9.2 kb, indicating some minor rearrangements. The median depth of coverage of the Tn125 transposon was 98.20× (compared to 83.0X for the whole genome). The alignment and annotation for the Tn125 transposon are visually represented in Fig. 1. An additional de novo assembly was then performed using plasmidSpades 3.13.0 with the ‘-plasmid’ and ‘-careful’ options enabled [22] to reconstruct putative plasmids. This resulted in 21 contigs, with the *bla*_NDM-1_ gene located near the center of the largest contig (88,063 bp) and its surrounding region of 7626 bp aligned to the Tn125 transposon with over 99% sequence identity. Outside of this region, no alignments were found between this putative plasmid contig and the pNDM-BJ01 (NCBI NC_019268.1) plasmid. Additional screening of the putative plasmid contig against the Plasmid Database (PLSDB) online platform [23] (v2020_03_04) with mash was therefore performed and provided four matches: a large plasmid of 78,125 bp (NCBI CP038501.1) that was found in *A. baumannii*, and three smaller ones (NCBI JQ739158.1, KF220658.1, KR059864.1) with sizes 4797, 1634 and 7865 bp, respectively. Alignments with BLAST showed that the majority of the putative plasmid contig carrying *bla*_NDM-1_ aligned to regions on the larger CP038501.1 plasmid (albeit with several rearrangements), except for the region containing the sequence that aligned to the Tn125 transposon as illustrated in Fig. 2. The three smaller plasmid matches all corresponded to parts of the Tn125 transposon (results not shown).

## Discussion and conclusion

To the best of our knowledge, we present the first description of a NDM-1 producing *A. baumannii* isolated in Benin. In particular, we detected the Tn125 transposon that harbors the *bla*_NDM-1_ gene on a contig of 88,063 bp. Because this contig was generated with plasmidSpades and a search against PLSDB demonstrated high similarity to the CP038501.1 plasmid previously reported in *A. baumannii* but not carrying the *bla*_NDM-1_ gene, our results indicate that most likely a plasmid similar to CP038501.1 obtained the *bla*_NDM-1_ gene through the integration of the Tn125 transposon. In particular, the horizontal transfer capacity of this transposon in relationship with genes encoding NDM has been previously demonstrated within *Acinetobacter* and also other bacteria [24, 25]. Moreover, the strain showed an XDR pattern with resistance against several other antibiotics confirmed both by phenotypical testing and the detection of several other resistance genes, thereby limiting therapeutic options. Accordingly, *A. baumannii* clinical isolates usually exhibit multidrug resistance phenotypes, facilitating their persistence in hospital settings [26]. The ease of availability of antibiotics is probably one of the biggest contributors to antibiotics resistance. Especially in developing countries, there is little regulation on the retail of pharmaceuticals. Moreover, the strain harbored several virulence genes related to biofilm formation. Biofilm formation in *A. baumannii* has been suggested to decrease the diffusion of drugs through the bacterial cells leading to multidrug resistance and also aids the strong survival ability *of A. baumannii* in harsh environments [26].

Low- and middle-income countries suffer a lack of infrastructure and resources to monitor AMR and perform optimal antibiotic treatment. For instance, cefazolin is the recommended antibiotic for surgical antimicrobial prophylaxis (SAP), but unfortunately this antibiotic is unavailable in Benin. The use of broad spectrum cephalosporins such as ceftriaxone in SAP is even more likely to induce resistance than cefazolin and other widely used surgical prophylactic drugs. Treatment options are limited for patients infected with XDR strains, increasing the severity of such infections. In this case, the XDR strain was only susceptible to amikacin, ciprofloxacin and colistin. Amikacin is however only available for one case out of two in Benin. Clinicians are often forced to buy it from neighboring countries such as Nigeria or Togo. Novel treatments effective against NDM-producing *A. baumannii* isolates are limited, and reduced activity was observed for strains that produced particular types of other beta-lactamases such as oxacillinases [27]. Moreover, Benin does not have the necessary quality assurance mechanisms to ensure that antibiotics being supplied are of high quality. Knowledge of the occurrence of NDM-1 producing bacteria may encourage pharmaceutical companies and the Ministry of Health to facilitate the provision of ‘last-resort’ antibiotics. Innovative therapeutic strategies such as bacteriophage therapy and monoclonal antibodies, or soon-to-be commercially available antibiotics such as plazomicin or cefiderocol, should also be considered for future use [27].

The threat of increasing antibiotics resistance is not limited to *A. baumannii*. For instance, methicillin-resistant *Staphylococcus aureus* and extended-spectrum beta-lactamase producing organisms are also frequently detected in certain public teaching hospitals. Our laboratories need to be better resourced so that they can deliver antibiotics susceptibility information. This includes equipment, consumable resources and their supply chain, as well as development of the necessary human resources to perform tests, store, curate and disseminate data [28]. Unfortunately, phenotypic detection of carbapenemase-producing bacteria is not routinely performed. The immunochromatographic RESIST-3 O.K.N. ICT test that can detect the presence of NDM enzymes, represents a cost-effective alternative to costlier and less widely available characterization methods that rely on molecular amplification.

In conclusion, the isolation of this XDR *A. baumannii* strain containing genes encoding NDM-1 and other beta-lactamases is worrying. The presence of *bla*_NDM-1_ located on the Tn125 transposon integrated in a plasmid represents a serious threat because of its potentially high horizontal transferability. Carbapenemase-producing XDR bacteria can be fatal in resource limited countries where therapeutic antibiotic options are restricted. Through this study, we want to alarm the official and governmental agencies to contribute to strengthening laboratory services and equipment at both the local and national level, and urge them to make arrangements for the provision of drugs such as avibactam combinations in Benin. Additionally, further efforts to characterize the molecular structure of this plasmid will aid to assess its transferability and hence risk of spread in the overall population, as well as benefit developing targeted detection methods for surveillance at the population level.

## Supplementary information

**Additional file 1.** Alignment results for all detected antimicrobial resistance gene (NCBI-AMR).

**Additional file 2.** Virulence factor database (VFDB) of *A.baumannii* NDM-1.

## Data Availability

Generated WGS data in this study have been submitted to NCBI SRA as BioProject PRJNA624101. The Additional file 2 consists out of detailed alignment results for all detected antimicrobial resistance (‘gene_detection-NCBI_AMR.zip’) and virulence factor genes (‘gene_detection-VFDB_full.zip’).

## Abbreviations

EUCAST: European Committee on Antimicrobial Susceptibility Testing
MDR: Multidrug-resistant
NDM-1: New-Delhi metallo-beta-lactamase-1
VFDB: Virulence factor database
ST: Sequence type
XDR: Extensively drug-resistant
PLSDB: Plasmid database
