# Supplementary material for: First detection of a plasmid-encoded New-Delhi metallo-beta-lactamase-1 (NDM-1) producing Acinetobacter baumannii using whole genome sequencing, isolated in a clinical setting in Benin
Source: Ann Clin Microbiol Antimicrob. 2021 Jan 6;20:5. doi: 10.1186/s12941-020-00411-w (PMC7789245; doi:10.1186/s12941-020-00411-w)
Supplement: Supplementary file 2 — Additional file 2. Virulence factor database (VFDB) of A.baumannii NDM-1. [file 12941_2020_411_MOESM2_ESM.zip › gene_detection-NCBI_AMR/gene_detection-3159.html]

Gene detection: Sample(name='3159', run='2019\_071', fq\_fwd=PosixPath('/scratch/bebog/wgs\_liege/input/2019\_071/3159\_S2\_L001\_R1\_001.fastq.gz'), fq\_rev=PosixPath('/scratch/bebog/wgs\_liege/input/2019\_071/3159\_S2\_L001\_R2\_001.fastq.gz'), metadata={'name': '3159', 'id': '3159', 'species': 'A. baumannii', 'species\_short': 'abaumannii'}) 

### NCBI AMR genes

| Locus | Length | % Covered | Mismatches | Uncertainty | Divergence (%) | Depth | Accession |
| --- | --- | --- | --- | --- | --- | --- | --- |
| aac(3)-IId | 1061 | 93.78 | 66holes | edge0.0 | 0.00 | 128.20 | - |
| ant(3'')-IIa | 989 | 100.00 | 7snp1indel | - | 0.71 | 51.11 | - |
| aph(3'')-Ib | 1028 | 90.08 | 102holes | edge0.0 | 0.00 | 83.06 | - |
| aph(6)-Id | 1037 | 100.00 | - | - | 0.00 | 98.96 | - |
| blaADC-166 | 1152 | 100.00 | 18snp | - | 1.56 | 42.16 | - |
| blaNDM-1 | 1013 | 100.00 | - | - | 0.00 | 101.37 | - |
| blaOXA-558 | 825 | 100.00 | 1snp | - | 0.12 | 54.23 | - |
| blaOXA-58 | 1043 | 100.00 | - | - | 0.00 | 115.58 | - |
| ble | 566 | 100.00 | - | - | 0.00 | 119.86 | - |
| mph(E) | 1085 | 100.00 | - | - | 0.00 | 138.39 | - |
| msr(E) | 1676 | 100.00 | - | - | 0.00 | 167.73 | - |
| sul2 | 1016 | 91.53 | 2snp86holes | edge0.0 | 0.21 | 66.53 | - |
| tet(39) | 1388 | 98.56 | 3snp20holes | edge0.0 | 0.22 | 148.26 | - |

  
Download (TSV)  

Last updated: 23-11-2018
